# OP47 Improving The Reporting Of Horizon Scanning Methods: Developing A Checklist Of Preferred Reporting Items

**DOI:** 10.1017/S0266462325101050

**Published:** 2025-12-29

**Authors:** Sonia Garcia Gonzalez-Moral, Sarah Khan, Ross Fairbairn, Oleta Williams, Megan Fairweather, Gill Norman

## Abstract

**Introduction:**

Horizon scanning (HS) systematically identifies emerging health technologies to support future decision-making. However, HS methods face challenges due to inconsistent terminology and lack of reporting standards. This hinders dissemination and increases research waste. Unlike PRISMA for evidence synthesis, no reporting guidelines exist to support HS. This work proposed a preliminary reporting checklist.

**Methods:**

In July 2024, a working group comprising HS analysts, evidence synthesis researchers, and information specialists was established at the Innovation Observatory (IO), the UK’s national HS organization. The group convened three times to define objectives, refine methods, and develop the reporting checklist. In the first meeting, we outlined a prototype by adapting the PRISMA Extension for Scoping Reviews (PRISMA-ScR). We internally validated it on 25 IO HS reports (2017 to 2024). The second meeting assessed the relevance of each checklist item following our internal validation. In the third meeting, additional items were proposed based on the validation’s results, and the group reached consensus on the final checklist.

**Results:**

The final checklist prototype comprised a total of 35 items of which 29 were essential and six optional. It introduced new key elements absent in PRISMA-ScR: “interest holder description” in the introduction, “horizon-scanning scope” in the methods, “technology characteristics” in the results and “political, economic, societal, technological, legal and environmental (PESTLE) interpretation” in the discussion. Additionally, the checklist was tailored to promote transparency, equity, fairness, and sustainability in signal detection processes over those characteristics in evidence synthesis, aligning with the goals of HS. The checklist is available to download in the Open Science Framework.

**Conclusions:**

Our checklist addressed a critical methodological gap, advancing HS research by promoting standardization and transparency of HS methods. By making the draft checklist available, we encourage HS practitioners to pilot it. Feedback is welcomed to contribute to the final checklist. Its adoption and endorsement will improve output visibility, ensuring this meets necessary methodological standards underpinning unbiased healthcare policy decision-making.

